# Ligand-Based Virtual Screening Based on the Graph Edit Distance

**DOI:** 10.3390/ijms222312751

**Published:** 2021-11-25

**Authors:** Elena Rica, Susana Álvarez, Francesc Serratosa

**Affiliations:** Departament d’Enginyeria Informàtica i Matemàtiques, Universitat Rovira i Virgili, 43007 Tarragona, Spain; susana.alvarez@urv.cat (S.Á.); francesc.serratosa@urv.cat (F.S.)

**Keywords:** virtual screening, molecular similarity, extended reduced graph, structure activity relationships, machine learning, graph edit distance

## Abstract

Chemical compounds can be represented as attributed graphs. An attributed graph is a mathematical model of an object composed of two types of representations: nodes and edges. Nodes are individual components, and edges are relations between these components. In this case, pharmacophore-type node descriptions are represented by nodes and chemical bounds by edges. If we want to obtain the bioactivity dissimilarity between two chemical compounds, a distance between attributed graphs can be used. The Graph Edit Distance allows computing this distance, and it is defined as the cost of transforming one graph into another. Nevertheless, to define this dissimilarity, the transformation cost must be properly tuned. The aim of this paper is to analyse the structural-based screening methods to verify the quality of the Harper transformation costs proposal and to present an algorithm to learn these transformation costs such that the bioactivity dissimilarity is properly defined in a ligand-based virtual screening application. The goodness of the dissimilarity is represented by the classification accuracy. Six publicly available datasets—CAPST, DUD-E, GLL&GDD, NRLiSt-BDB, MUV and ULS-UDS—have been used to validate our methodology and show that with our learned costs, we obtain the highest ratios in identifying the bioactivity similarity in a structurally diverse group of molecules.

## 1. Introduction

The high increase in chemical compounds data has created the need to develop computational tools to reduce the drug synthesis and drug test cycle runtimes. When activity data are analysed, these tools are required to generate new models for virtual screening techniques [1,2,3]. In the drug discovery process, virtual screening is a common step in which computational techniques are used to search and filter chemical compounds in databases. Basically, there are two main types of methods in the virtual screening: ligand-based virtual screening (LBVS) [4] and structure-based virtual screening (SBVS) [5]. In this work, we focus only in LBVS applications. The idea of the LBVS method is to predict the unknown activity of new molecules [6] using the information about the known activity of some molecules—specifically, their behaviour as ligands that bind to a receptor.

Some LBVS approaches are shape-based similarity [7], pharmacophore mapping [6], fingerprint similarity and machine learning methods [8]. According to [9], structurally similar molecules are presumed to have similar activity properties, then, in the context of LBVS methods, the chosen molecular similarity metric is important because it can determine the success of a virtual screening method to discover proper drug candidates. Various similarity methods are used in several applications [10,11,12,13,14].

To compute molecular similarity, it is necessary to define a distance and define a descriptor representing the molecule. Hundreds of molecular descriptors have been reported in the literature [15]. For instance, one-dimensional descriptors include general molecular properties, such as size, molecular weight, logP or dipole moment, or BCUT parameters [16,17,18,19]. Two-dimensional descriptors generate an array of representations of the molecules by simplifying the atomic information within them, such as 2D fingerprints [20,21,22]. Finally, three-dimensional descriptors use 3D information, such as molecular volume [23,24]. Other existing methods, instead of representing molecules by an N-dimensional vector, use relational structures, such as trees [25] or graphs [26,27]. Regarding the molecule representation by graphs, some methods represent compounds using reduced graphs [28,29,30,31] and other ones, such as extended reduced graphs (ErGs) [28]. Reduced graphs group atomic sub-structures that have related features, e.g., pharmacophoric features, ring systems, hydrogen-bonding or others. Moreover, ErGs are an extension of reduced graphs that introduce some changes to better represent shape, size and pharmacophoric properties of the molecules. The method presented in [28] has demonstrated its use as a powerful tool for virtual screening.

To perform reduced graph comparisons, three different similarity measures have been used: In [28,29,32], they map the reduced graphs into a 2D fingerprint. In [33], they map reduced graphs into sets of shortest paths. Finally, in [34,35], they perform the comparison on the graphs using the Graph Edit Distance (GED). GED considers the distance between two graphs as the minimum cost of modifications required to transform one graph into another. Each modification can be one of the following six operations: insertion, deletion and substitution of both nodes and edges in the graph [36,37,38]. The main goal of this paper is to present an algorithm that learns the edit costs in the GED to improve the classification ratio returned by the system when the Harper costs were used.

In an initial paper [34], the edit costs were imposed and extracted from [33], given the chemical expertise of the authors and considering the different node and edge types. Later, in [35], authors presented an algorithm for optimising those edit costs based on minimising the distance between correctly classified molecules and maximising the distance between incorrectly classified molecules. That work was inspired in a similar one carried out by Birchall et al. [39], in which the authors optimise the transformation costs of a String Edit Distance method to compare molecules using reduced graphs.

The main problem of the algorithm in [35] was the huge computational cost, which depends on the number of edit costs to be optimised. Thus, for practical reasons, in the experimental section in [35], they presented four experiments, in which only one edit cost was optimised in each experiment. They imposed the other costs (126 in total) to be the ones defined in [33]. In contrast, starting from the costs defined by [33], our method learns the whole edit costs of the GED to compare molecules with a lower computational cost obtaining higher classification ratios in the ligand-based screening application, as shown in the experimental section.

The outline of this paper is as follows. First, materials and methods are explained in detail, including the datasets, the GED and the learning algorithm. Second, the results of the practical experiments are described and discussed. Third and finally, a general discussion about the method and the results is presented.

## 2. Materials and Methods

### 2.1. Datasets

In this study, we have used six available public datasets: ULS-UDS [40], GLL&GDD [41], CAPST [42], DUD-E [43], NRLiSt-BDB [44] and MUV [45]. All these datasets had been formatted and standardized by the LBVS benchmarking platform developed by Skoda and Hoksza [46]. The datasets are composed of various groups of active and inactive molecules arranged according to the purpose of a target. Each group is split in two halves, the test and train sets, which are required when using machine learning methods. The train set is used to optimize the transformation costs, and the test set is used to evaluate the classification ratio. The targets of the datasets are shown in Table 1. In our experimentation, we have taken a subset of the first 100 active molecules and 100 of the first inactive molecules per target. Some datasets have less than 100 active molecules; in this case, all active molecules are taken and also the same number of inactive molecules.

### 2.2. Molecular Representation

**Figure 1:**
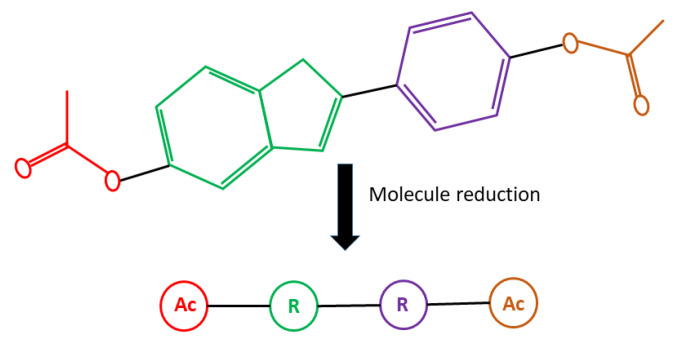
Example of molecule reduction using ErG. The original molecule is on the top and its ErG representation is below. Elements of the same colour on the top are reduced to nodes on the ErG. R: Ring system, Ac: Acyclic components.

Reduced graphs are compact representations of chemical compounds, in which the main information is condensed in feature nodes to give abstractions of the chemical structures. Different versions of reduced graphs have been presented [26,28,30,32,33] that depend on the features they summarise or the use that is given to them. In the virtual screening context, the structures are reduced to track down features or sub-structures that have the potential to interact with a specific receptor and, at the same time, try to keep the topology and spatial distribution of those features. Figure 1 presents an example of molecule reduction.

### 2.3. The Proposed Method

We explain our proposed method in the next three subsections. The first one explains the classification of compounds based on structural information; in the second one, we explain the learning algorithm; and in the third one, we detail the code of the algorithm.

#### 2.3.1. Classification of Molecules

Once the molecules are represented as ErGs, we can compare them by means of the Graph Edit Distance (GED) [47,48]. The GED is defined as the minimum cost of transformations required to convert one graph into the other. Thus, in our application, it is the cost to transform an ErG into the other one. These modifications are called edit operations, and six of them have been defined: insertion, deletion and substitution of both nodes and edges. To classify a molecule, we apply the Nearest Neighbour (NN) strategy that consists of calculating the GED between this molecule and the other ones, which we know their class, and predicting its class (active or inactive) to be the class of the nearest molecule. In the case the molecule is equidistant from more than one classified molecule, the method arbitrarily selects one of the closest molecules.

Edit costs have been introduced to quantitatively evaluate each edit operation. The aim of the edit costs is to designate a coherent transformation penalty in proportion to the extent to which it modifies the transformation sequence. For instance, when ErGs are compared, it makes sense that the cost of substituting a “hydrogen-bond donor" feature with a joint “hydrogen-bond donor-acceptor" feature be less heavily penalized than the cost of substituting a “hydrogen-bond donor" feature with an “aromatic ring" system. Similarly, inserting a single bond should have a lower penalization cost than inserting a double bond, and so on. In a previous work [34], the edit costs proposed by Harper et al. [33] were used. The node and edge descriptions are shown in Table 2, and the specific costs proposed by Harper et al. are exposed in Table 3 and Table 4.

**Figure 2:**
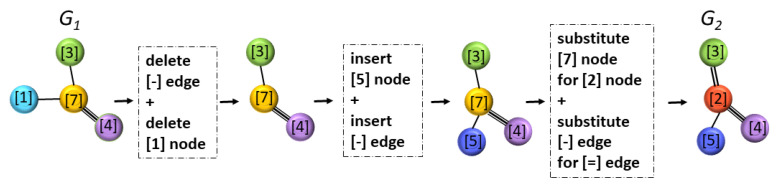
Transformation sequence from graph $G_{1}$ to graph $G_{2}$.

The final edit cost for a given transformation sequence is obtained by adding up all of the individual edition costs. Figure 2 shows a schematic example of a transformation of a molecule $G_{1}$ into another one, $G_{2}$. As we can see, the executed operations in this transformation are: a deletion of node type [1], a deletion of a simple edge, an insertion of node type [5], an insertion of a simple edge a substitution of node type [7] by node of type [2], and a substitution of a simple edge with a double edge. If we sum the values of Harper costs associated with these operations in Table 3 and Table 4, we obtain that the cost of this transformation equals: 2+0+2+0+3+3=10.

Since several transformation sequences can be applied to transform a graph into another one, the GED resulting for any pair of graphs is defined as the minimum cost under all those possible transformation sequences. Usually, the final distance is normalized according to the number of nodes in both graphs being compared. This is performed in order to make the measure independent of the size of the graphs.

More formally, we define the GED as follows,

(1) $$GED\left(G_{a},G_{b},C_{1},\ldots ,C_{n}\right)=\min_{\left\{N_{i}:i=1,\ldots ,n\right\}}\frac{C_{1}N_{1}+\ldots +C_{n}N_{n}}{L}$$

where $C_{t}$ is the imposed cost of the tth edit operation on nodes and edges, and $N_{t}$ is the number of times this edit operation has been applied. Moreover, the combination of $N_{1}$, $N_{2},\ldots$ is restricted to be one that transforms $G_{a}$ into $G_{b}$. Finally, *L* is the sum of the number of nodes of both graphs, and *n* is the number of different edit operations on nodes and edges.

Several GED computational methods have been proposed during the last three decades, they can be classified into two groups: those returning the exact value of the GED in the exponential computational cost with respect to the number of nodes [49], and those returning an approximation of the GED in the polynomial cost [50,51,52,53]. These two groups of GED computational methods have been widely studied [54,55]. In our experiments, we used the fast bipartite graph matching method [50] (polynomial computational cost), although our learning method is independent of the matching algorithm.

Initially, the edit costs were manually set in a trial and error process considering the application at hand [33,34]. (As previously commented, Table 3 and Table 4 show their edit cost proposal.) Nevertheless, there has been a tendency to automatically learn these costs since it has been seen that a proper tuning of them is crucial to achieve good classification ratios in virtual screening [35] and other applications [56,57,58,59,60]. In [35], authors presented a learning algorithm that is forced to learn only one edit cost at once due to runtime restrictions. Thus, they perform four different experiments on the same data as [34] in which they use all the costs of [34] except the one that is learned. These experiments are: C1: Learning the deletion/insertion cost of the carbon link ([6]). C2: Learning the cost of substituting a carbon link node ([6]) with an aromatic ring system ([5]). C3: Learning the insertion/deletion cost of the bond edge ([-]). C4: Learning the substitution cost between a single bond edge ([-]) and a double bond edge ([=]). Table 5 shows their learnt costs.

The next section presents our method, which has the advantage of learning the whole set of edit costs at once.

#### 2.3.2. The Learning Method

We present an iterative algorithm, in which, in each iteration, the NN strategy is applied and the initial edit costs are modified such that one molecule that has been incorrectly classified becomes correctly classified. Modifying the edit costs could cause other incorrectly classified molecules to also be properly classified, but, unfortunately, some other ones that were properly classified become incorrectly classified. This is the reason why we want to generate the minimum modification on the edit costs. To do so, the selected molecule is the one that it is easier to move from the incorrectly classified ones to the correctly classified ones. In the next paragraphs, our learning algorithm is explained in detail.

Let $G_{j}$ be a molecule in the learning set that has been incorrectly classified using the NN strategy and the current costs $C_{1},\ldots ,C_{n}$. We define $D_{j}$ as the minimal GED between $G_{j}$ and all the molecules but restricted to be the ones that have a different class:

(2) $$D_{j}=\min_{q}GED\left(G_{j},G_{q},C_{1},\ldots ,C_{n}\right)\;,\;\mathrm{where}\;\mathrm{class}(G_{q})\;\neq \;\mathrm{class}(G_{j}).$$

Moreover, we define $D_{j}^{\prime }$ as the minimal GED between $G_{j}$ and all the molecules of the learning set but restricted to be the ones that have the same class:

(3) $$D_{j}^{\prime }=\min_{p}GED\left(G_{j},G_{p},C_{1},\ldots ,C_{n}\right),\;\mathrm{where}\;\mathrm{class}(G_{p})\;=\;\mathrm{class}(G_{j})$$

**Figure 3:**
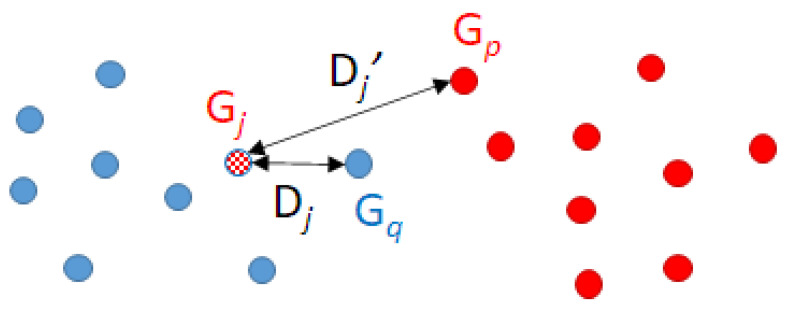
Classification of molecule $G_{j}$. The true classes are in solid colours. $G_{j}$ is classified in the wrong class (blue), but the correct class is the red one. The distance between $G_{j}$ and $G_{q}$ is lower than the distance between $G_{j}$ and $G_{p}$.

Since $G_{j}$ is incorrectly classified, we can confirm that $D_{j}^{\prime }>D_{j}$. Figure 3 schematically shows this situation. It turns out that $G_{j}$ and $G_{q}$ belong to different classes even though the distance between them is smaller than the distance between $G_{j}$ and its closest molecule that has the same class, $G_{p}$.

The main idea of our method is to permute $D_{j}^{\prime }$ and $D_{j}$, modifying the edit costs. With this exchange, we achieve a lower distance between $G_{j}$ and the molecule of its same class ($G_{p}$) than the distance between $G_{j}$ and the molecule with different classes ($G_{q}$). Thus, $G_{j}$ will be correctly classified. However, considering that adapting these distances affects all the molecules’ classifications, we select a molecule $G_{i}$ among the incorrectly-classified ones, $\{G_{j}|D_{j}^{\prime }>D_{j}$, $\forall G_{j}\}$, which satisfies that the difference of the distances $D_{j}^{\prime }-D_{j}$ is the minimum one, as shown in Equation (4). Note that in Equation (4), all the values of $D_{j}^{\prime }-D_{j}$ are always positive because $D_{j}^{\prime }>D_{j}$ by definition of $G_{j}$.

(4) $$G_{i}=arg\min_{\left\{G_{j}|D_{j}^{\prime }>D_{j}\right\}}\left(D_{j}^{\prime }-D_{j}\right)$$

**Figure 4:**
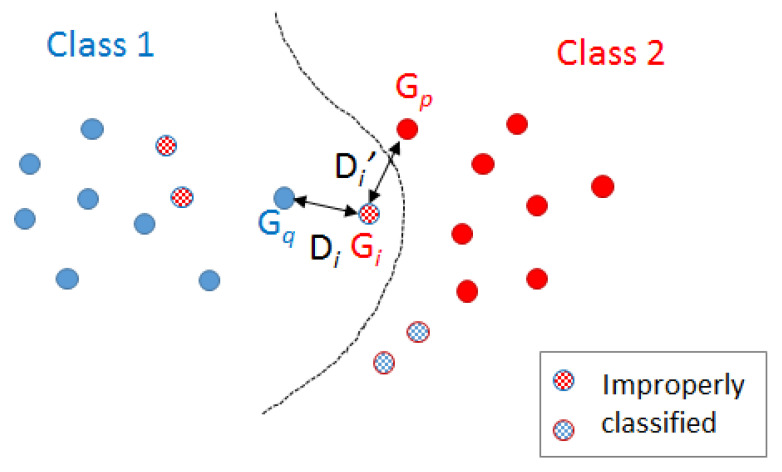
Stripped molecules have been improperly classified using NN strategy. $G_{i}$ is the one that minimises $D_{j}^{\prime }-D_{j}$ being $D_{j}^{\prime }>D_{j}$.

Figure 4 shows this idea. However, what is crucial to understand is that this modification is performed in the distances since the molecule representations are not modified. Furthermore, this is carried out by modifying the edit costs. Thus, the strategy is to define the new edit costs such that $D_{i}^{\prime }$ becomes $D_{i}$ and vice versa.

The rest of this section is devoted to explaining how to modify the edit costs.

Considering Equation (1), the distance is composed of edit costs $C_{1},\ldots ,C_{n}$ and the number of times the specific edit operations have been taken $N_{1},\ldots ,N_{n}$. Our method modifies the edit costs without altering the number of operations $N_{1},\ldots ,N_{n}$.

Thus, we define $D_{i}$ and $D_{i}^{\prime }$ as follows:

(5) $$\begin{array}{c} D_{i}=\frac{C_{1}N_{1}+\ldots +C_{n}N_{n}}{L}\\ D_{i}^{\prime }=\frac{C_{1}N_{1}^{\prime }+\ldots +C_{n}N_{n}^{\prime }}{L^{\prime }} \end{array}$$

Then, we exchange the distances $D_{i}$ and $D_{i}^{\prime }$ and modify the edits costs by adding new terms:

(6) $$\begin{array}{c} D_{i}=\frac{\left(C_{1}+\alpha_{1}^{\prime }\right)N_{1}^{\prime }+\ldots +\left(C_{n}+\alpha_{n}^{\prime }\right)N_{n}^{\prime }}{L^{\prime }}\\ D_{i}^{\prime }=\frac{\left(C_{1}+\alpha_{1}\right)N_{1}+\ldots +\left(C_{n}+\alpha_{n}\right)N_{n}}{L} \end{array}$$

Note that these new terms $\alpha_{1},\ldots ,\alpha_{n}$ and also $\alpha_{1}^{\prime },\ldots ,\alpha_{n}^{\prime }$ are defined such that the new value of $D_{i}$ is $D_{i}^{\prime }$ instead of $D_{i}$ and vice versa. Moreover, the edit costs $C_{1},\ldots ,C_{n}$ are the same in both expressions. We proceed to explain below how to deduce the terms $\alpha_{1},\ldots ,\alpha_{n}$ and also $\alpha_{1}^{\prime },\ldots ,\alpha_{n}^{\prime }$.

From Equation (6), we obtain:

(7) $$\begin{array}{c} D_{i}=\frac{C_{1}N_{1}^{\prime }+\ldots +C_{n}N_{n}^{\prime }}{L^{\prime }}+\frac{\alpha_{1}^{\prime }N_{1}^{\prime }+\ldots +\alpha_{n}^{\prime }N_{n}^{\prime }}{L^{\prime }}\\ D_{i}^{\prime }=\frac{C_{1}N_{1}+\ldots +C_{n}N_{n}}{L}+\frac{\alpha_{1}N_{1}+\ldots +\alpha_{n}N_{n}}{L} \end{array}$$

We observe that the first terms in both expressions are $D_{j}^{\prime }$ and $D_{j}$, respectively:

(8) $$\begin{array}{c} D_{i}=D_{i}^{\prime }+\frac{\alpha_{1}^{\prime }N_{1}^{\prime }+\ldots +\alpha_{n^{\prime }}^{\prime }N_{n}^{\prime }}{L^{\prime }}\\ D_{i}^{\prime }=D_{i}+\frac{\alpha_{1}N_{1}+\ldots +\alpha_{n}N_{n}}{L} \end{array}$$

By regrouping the terms again, we have:

(9) $$\begin{array}{c} D_{i}-D_{i}^{\prime }=\frac{\alpha_{1}^{\prime }N_{1}^{\prime }+\ldots +\alpha_{n^{\prime }}^{\prime }N_{n}^{\prime }}{L^{\prime }}\\ D_{i}^{\prime }-D_{i}=\frac{\alpha_{1}N_{1}+\ldots +\alpha_{n}N_{n}}{L} \end{array}$$

Furthermore, finally, we divide by $D_{i}-D_{i}^{\prime }$ and $D_{i}^{\prime }-D_{i}$ in each expression to arrive at the following normalised expressions:

(10) $$\begin{array}{c} 1=\frac{\alpha_{1}^{\prime }N_{1}^{\prime }}{\left(D_{i}-D_{i}^{\prime }\right)L^{\prime }}+\ldots +\frac{\alpha_{n}^{\prime }N_{n}^{\prime }}{\left(D_{i}-D_{i}^{\prime }\right)L^{\prime }}\\ 1=\frac{\alpha_{1}N_{1}}{(D_{i}^{\prime }-D_{i})L}+\ldots +\frac{\alpha_{n}N_{n}}{(D_{i}^{\prime }-D_{i})L} \end{array}$$

Note that, as commented in the definition of the GED, not all of the edit operations are used to transform a molecule into another. These edit operations are the ones that $N_{t}=0$ or $N_{t}^{\prime }=0$. Because of this, in Equation (10), there are some addends that are null. We use *m* and $m^{\prime }$ to denote the number of edit operations that have been used, that is, the ones that $N_{t}\neq 0$ or $N_{t}^{\prime }\neq 0$, respectively.

We want to deduce $\alpha_{1},\alpha_{2},\ldots$ and also $\alpha_{1}^{\prime },\alpha_{2}^{\prime },\ldots$ such that Equation (10) is fulfilled. The easiest way is to impose that each non-null term in these expressions equal $1/m^{\prime }$ or 1/m, respectively. Then, we achieve the following expressions,

(11) $$\begin{array}{c} 1/m^{\prime }=\frac{\alpha_{t}^{\prime }N_{t}^{\prime }}{\left(D_{i}-D_{i}^{\prime }\right)L^{\prime }}\;\mathrm{being}\;N_{t}^{\prime }>0\\ 1/m=\frac{\alpha_{t}N_{t}}{(D_{i}^{\prime }-D_{i})L}\;\mathrm{being}\;N_{t}>0 \end{array}$$

From the previous expressions, we arrive at the definitions of $\alpha_{t}$ that allow the modification from $D_{i}$ to $D_{i}^{\prime }$. Moreover, we also arrive at the definitions of $\alpha_{t^{\prime }}^{\prime }$ that allow the modification from $D_{i}^{\prime }$ to $D_{i}$.

(12) $$\begin{array}{c} \alpha_{t}^{\prime }=\frac{\left(D_{i}-D_{i}^{\prime }\right)L^{\prime }}{m^{\prime }N_{t^{\prime }}^{\prime }},N_{t}^{\prime }>0\\ \alpha_{t}=\frac{(D_{i}^{\prime }-D_{i})L}{mN_{t}},N_{t}>0 \end{array}$$

Note that considering Equations (5), (6) and (12), we have, on one hand, that the new costs ${\bar{C}}_{t}=C_{t}+\alpha_{t}$ and, on the other hand, that ${\bar{C}}_{t}=C_{t}+\alpha_{t}^{\prime }$. Since it may happen that $\alpha_{t}\neq \alpha_{t}^{\prime }$, we assume the average option is the best choice when both weights are computed,

(13) $${\bar{C}}_{t}=\left\{\begin{array}{c} C_{t}+\frac{\alpha_{t}+\alpha_{t}^{\prime }}{2},\;\mathrm{if}\;N_{t}>0\;\mathrm{and}\;N_{t}^{\prime }>0\\ C_{t}+\alpha_{t},\;\mathrm{if}\;N_{t}>0\;\mathrm{and}\;N_{t}^{\prime }=0\\ C_{t}+\alpha_{t}^{\prime },\;\mathrm{if}\;N_{t}=0\;\mathrm{and}\;N_{t}^{\prime }>0\\ C_{t},\;\mathrm{if}\;N_{t}=0\;\mathrm{and}\;N_{t}^{\prime }=0 \end{array}\right.$$

In the next subsection, we present our algorithm.

#### 2.3.3. Algorithm

Algorithm 1 consists of an iterative process in which, in each iteration, the edit costs are updated to correct the classification of one selected molecule. The updated costs are used in the next iteration to classify all the molecules again, select a molecule and modify the costs again.
**Algorithm 1** Costs learning.**Input (**Learning Set, Initial edit costs, Max_Iter**)** **Output (**Learnt edit costs**)** **Initialise:**iter=1.$C_{1}$, …, $C_{n}$ = Initial edit costs.**While**iter≤Max_Iter:  **Classify** all molecules with nearest neighbour and GED (Equation (1)) using $C_{1},\ldots ,C_{n}$.  **Compute $D_{j}$ and $D_{j}^{\prime }$**: (Equations (2) and (3)) for all $G_{j}$ incorrectly classified.  **Deduce** $G_{i}$ (Equation (4)).  **Compute $\alpha_{t}$, t=1,…,m and $\alpha_{t}^{\prime }$, $t=1,\ldots ,m^{\prime }$:** (Equation (12)).  **Compute** ${\bar{C}}_{1}$, …, ${\bar{C}}_{n}$ (Equation (13)).  **Update costs:** $C_{t}={\bar{C}}_{t},t=1,\ldots ,n$.  iter=iter+1. **End While****End Algorithm**

This algorithm has been coded in Matlab, and it is available in https://deim.urv.cat/~francesc.serratosa/SW/, accessed on 12 November 2021.

## 3. Results

Table 6 shows the classification ratios obtained in each dataset using different edit cost configurations, algorithms and initialisations. The first row corresponds to the accuracies obtained with the costs proposed by Harper [33], the second row corresponds to the accuracies deduced by setting all the costs to 1 (no learning algorithm). The next four rows correspond to the accuracies obtained using the costs deduced in García et al. [35] in their four experiments (C1, C2, C3 and C4). Finally, the last two rows present the accuracies obtained by our method: the first row by initialising the algorithm by the Harper costs and the second one by initialising all the costs to 1. We note the used costs are the mean of the learned costs in all the databases, and our algorithm performed 50 iterations.

We realise that in all the datasets, except for MUV and ULS-UDS, our costs with Harper initialisation obtained the highest classification ratios. In these two datasets, the best accuracy is obtained by Harper costs. Note that our method initialised by all-ones costs returns lower accuracies than our method initialised by Harper costs, except for the ULS-UDS dataset. This behaviour makes us think that the initialisation point is very important in this type of algorithm. Another highlight is that we have achieved better accuracies than the four experiments presented by García et al. [35] in all the tests. In the ULS-UDS dataset, our method returns close accuracy to the Harper costs. Nevertheless, that is not the case for MUV dataset. To deeply analyse this behaviour, we have computed the accuracy using the costs learned by only the MUV targets. In this case, the accuracy is 64.9%, which is significantly lower than using mean costs. This is not the normal behaviour in learning algorithms since while conducting specific learning, the classification ratio tends to increase. We think there are other reasons for this abnormal behaviour: one could be the small size of this dataset and the other the separability between ligands and decoys in MUV is low, which makes our algorithm not to converge to a proper solution.

**Figure 5:**
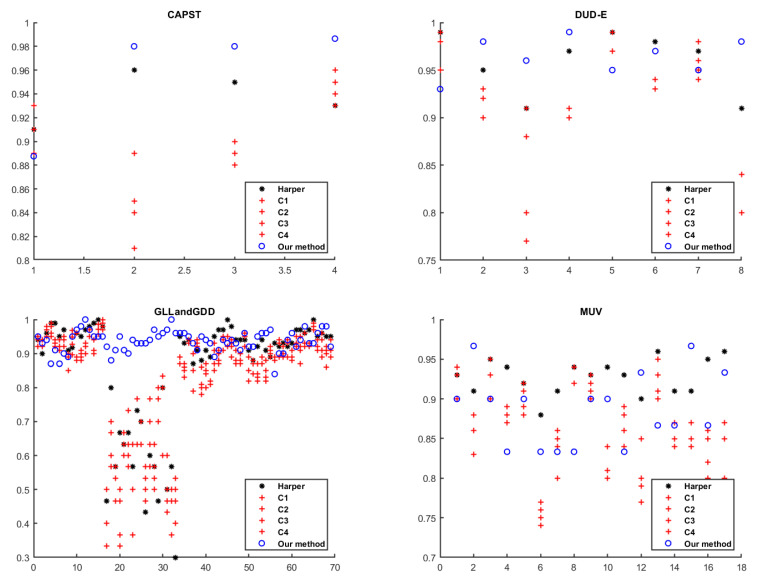

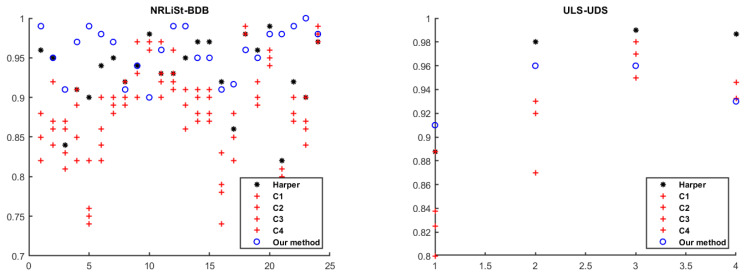
Classification ratio in the test set over the 127 targets available in the six datasets. The horizontal axis represents the index of the targets presented in Table 1.

In Figure 5, we present the classification ratio obtained in the 127 targets in the six datasets. At a glance, we realise that our method achieves most of the highest accuracies in all the targets in CAPST, DUD-E, GLL&GDD and NRLiSt-BDB databases. Specifically, we point out targets from 19 to 31 in the GLL&GDD dataset where the other cost combinations have very low accuracies while our method achieves much higher results. We observe that targets in the datasets MUV and ULS-UDS, in which our method does not return the highest accuracies, have a high variability because the same costs produce very different results.

**Figure 6:**
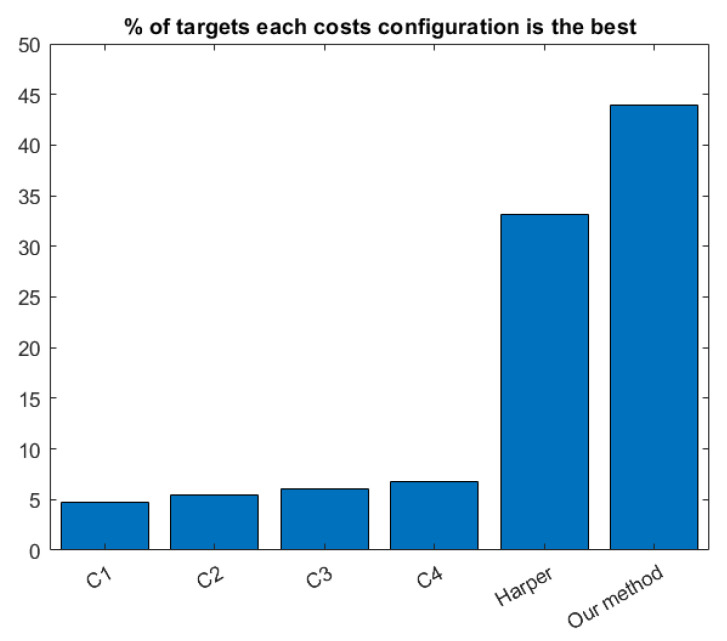
Percentage of times that each set of costs returns the best classification ratio.

Note that in [35], authors computed a cost per each of the six datasets and each target. Conversely, we learn the edit costs given the six datasets at once. In general, using several datasets at once makes the learnt parameters less specific for the application at hand, and thus, the classification ratios tend to decrease. In spite of this possible disadvantage, our method returns better classification ratios than the one in [35] in all the datasets. Figure 6 shows the percentage of times that each cost configuration obtains the highest classification ratio taking into account all the 127 targets, given the four configurations proposed in [35], one configuration proposed in [33] and our deduced configuration. Our method obtains the best classification ratio the highest number of times.

Table 7 and Table 8 show our learned edition costs for nodes and edges, respectively. In red and bold are the ones that are different to the ones proposed by Harper et al. [33]. As we can see, the results are very similar to Harper costs because we introduce a very small modification in each step. In addition, there are many costs that have not been modified. This is because these costs were not involved in the modifications of molecules that are improperly classified, minimising $D_{i}^{\prime }-D_{i}$.

## 4. Discussion

We present an iterative algorithm such that, in each iteration, the current costs are modified to properly classify an improperly classified molecule. While updating the costs, other improperly classified molecules could also be properly classified and vice versa. This is the reason why we cannot guarantee the algorithm’s convergence. To reduce the no-convergence impact and the possible solution oscillation, the algorithm selects the molecule that requires the minimum modification of the costs with the aim of slightly moving to the best solution.

The algorithm requires some initial costs. We have initialised the algorithm by some aleatory costs and by the costs proposed by Harper [33]. In all the tests, the highest accuracies appeared while initialising the costs by the Harper proposal. We believe this behaviour appears because the optimisation function of the learning algorithm is highly non-convex. Generally, in these situations, the selected initialisation has a high impact on the solution. Finally, we have seen that the classification accuracy is highly dependent on the edit costs. That is, a slight modification of one of the costs could make the classification be completely different. Considering that the computational cost of this learning problem is extremely high, sub-optimal algorithms, as the one presented, are needed to achieve an acceptable classification accuracy. Thus, any proposal that achieves better classification ratios would have to be considered and analysed.

## 5. Conclusions and Future Research

In some ligand-based virtual screening (LBVS) methods, molecules are represented by extended reduced graphs. In this case, the Graph Edit Distance can be used to compute the dissimilarity between molecules.

In this article, we have presented a new method that automatically learns the edit costs in the Graph Edit Distance. In each iteration, our method introduces slight modifications in the current costs to correct the classification of a selected molecule that had been incorrectly classified in the previous step.

The obtained costs have been tested in six publicly available datasets and have been compared to previous works published in [34,35]. We achieve better classification ratios than [35] in the six datasets and better classification ratios than [34] in four of them.

In the experimental section, we realised that small modifications in the costs could produce considerable improvement in the classification ratio.

In future work, we will analyse which types of molecules cause the algorithm to converge and which are the best initial values to obtain higher classification accuracy.

## Figures and Tables

**Table 1:** Datasets used for the experiments. Each dataset on the left contains the targets on the right.

Dataset	Used Targets
CAPST	CDK2, CHK1, PTP1B, UROKINASE
DUD-E	COX2, DHFR, EGFR, FGFR1, FXA, P38, PDGFRB, SRC, AA2AR
GLL&GDD	5HT1A_Agonist, 5HT1A_Antagonist, 5HT1D_Agonist, 5HT1D_Antagonist, 5HT1F_Agonist, 5HT2A_Antagonist, 5HT2B_Antagonist, 5HT2C_Agonist, 5HT2C_Antagonist, 5HT4R_Agonist, 5HT4R_Antagonist, AA1R_Agonist, AA1R_Antagonist, AA2AR_Antagonist, AA2BR_Antagonist, ACM1_Agonist, ACM2_Antagonist, ACM3_Antagonist, ADA1A_Antagonist, ADA1B_Antagonist, ADA1D_Antagonist, ADA2A_Agonist, ADA2A_Antagonist, ADA2B_Agonist, ADA2B_Antagonist, ADA2C_Agonist, ADA2C_Antagonist, ADRB1_Agonist, ADRB1_Antagonist, ADRB2_Agonist, ADRB2_Antagonist, ADRB3_Agonist, ADRB3_Antagonist, AG2R_Antagonist, BKRB1_Antagonist, BKRB2_Antagonist, CCKAR_Antagonist, CLTR1_Antagonist, DRD1_Antagonist, DRD2_Agonist, DRD2_Antagonist, DRD3_Antagonist, DRD4_Antagonist, EDNRA_Antagonist, EDNRB_Antagonist, GASR_Antagonist, HRH2_Antagonist, HRH3_Antagonist, LSHR_Antagonist, LT4R1_Antagonist, LT4R2_Antagonist, MTR1A_Agonist, MTR1B_Agonist, MTR1L_Agonist, NK1R_Antagonist, NK2R_Antagonist, NK3R_Antagonist, OPRD_Agonist, OPRK_Agonist, OPRM_Agonist, OXYR_Antagonist, PE2R1_Antagonist, PE2R2_Antagonist, PE2R3_Antagonist, PE2R4_Antagonist, TA2R_Antagonist, V1AR_Antagonist, V1BR_Antagonist, V2R_Antagonist
MUV	466, 548, 600, 644, 652, 689, 692, 712, 713, 733, 737, 810, 832, 846, 852, 858, 859
NRLiSt_BDB	AR_Agonist, AR_Antagonist, ER_Alpha_Agonist, ER_Alpha_Antagonist, ER_Beta_Agonist, FXR_Alpha_Agonist, GR_Agonist, GR_Antagonist, LXR_Alpha_Agonist, LXR_Beta_Agonist, MR_Antagonist, PPAR_Alpha_Agonist, PPAR_Beta_Agonist, PPAR_Gamma_Agonist, PR_Agonist, PR_Antagonist, PXR_Agonist, RAR_Alpha_Agonist, RAR_Beta_Agonist, RAR_Gamma_Agonist, RXR_Alpha_Agonist, RXR_Alpha_Antagonist, RXR_Gamma_Agonist, VDR_Agonist
ULS-UDS	5HT1F_Agonist, MTR1B_Agonist, OPRM_Agonist, PE2R3_Antagonist

**Table 2:** Node and edge attributes description in an ErG.

**Node Attributes**	
**Attribute**	**Description**
[0]	hydrogen-bond donor
[1]	hydrogen-bond acceptor
[2]	positive charge
[3]	negative charge
[4]	hydrophobic group
[5]	aromatic ring system
[6]	carbon link node
[7]	non-carbon link node
[0,1]	hydrogen-bond donor + hydrogen-bond acceptor
[0,2]	hydrogen-bond donor + positive charge
[0,3]	hydrogen-bond donor + negative charge
[1,2]	hydrogen-bond acceptor + positive charge
[1,3]	hydrogen-bond acceptor + negative charge
[2,3]	positive charge + negative charge
[0,1,2]	hydrogen-bond donor + hydrogen-bond acceptor + positive charge
**Edge Attributes**	
**Attribute**	**Description**
-	single bond
=	double bond
≡	triple bond

**Table 3:** Substitution, insertion and deletion costs for nodes proposed by Harper et al. [33].

**Substitution Costs for Nodes**															
	**[0]**	**[1]**	**[2]**	**[3]**	**[4]**	**[5]**	**[6]**	**[7]**	**[0, 1]**	**[0, 2]**	**[0, 3]**	**[1, 2]**	**[1, 3]**	**[2, 3]**	**[0, 1, 2]**
**[0]**	0	**2**	**2**	2	**2**	**2**	**2**	3	**1**	**1**	1	2	2	2	**1**
**[1]**	**2**	0	2	2	**2**	**2**	**2**	3	**1**	**2**	2	**1**	1	2	**1**
**[2]**	**2**	2	0	2	2	2	**2**	3	2	**1**	2	1	2	1	**1**
**[3]**	2	2	2	0	2	2	**2**	3	**2**	2	1	2	1	1	2
**[4]**	**2**	**2**	2	2	0	**2**	**2**	3	**2**	**2**	2	2	2	2	2
**[5]**	**2**	**2**	2	2	**2**	0	**2**	3	**2**	**2**	2	2	2	2	**2**
**[6]**	**2**	**2**	**2**	**2**	**2**	**2**	0	3	2	**2**	2	2	2	2	**2**
**[7]**	3	3	3	3	3	3	3	0	3	3	3	3	3	3	3
**[0, 1]**	**1**	**1**	2	**2**	**2**	**2**	2	3	0	**2**	2	2	2	2	**2**
**[0, 2]**	**1**	**2**	**1**	2	**2**	**2**	**2**	3	**2**	0	2	2	2	2	2
**[0, 3]**	1	2	2	1	2	2	2	3	2	2	0	2	2	2	2
**[1, 2]**	2	**1**	1	2	2	2	2	3	2	2	2	0	2	2	2
**[1, 3]**	2	1	2	1	2	2	2	3	2	2	2	2	0	2	2
**[2, 3]**	2	2	1	1	2	2	2	3	2	2	2	2	2	0	2
**[0, 1, 2]**	**1**	**1**	**1**	2	2	**2**	**2**	3	**2**	2	2	2	2	2	0
**Insertion/Deletion Costs for Nodes**															
	**[0]**	**[1]**	**[2]**	**[3]**	**[4]**	**[5]**	**[6]**	**[7]**	**[0, 1]**	**[0, 2]**	**[0, 3]**	**[1, 2]**	**[1, 3]**	**[2, 3]**	**[0, 1, 2]**
**insert**	**2**	**2**	2	2	**2**	**2**	**1**	1	**2**	**2**	2	**2**	2	2	**2**
**delete**	**2**	**2**	2	2	**2**	**2**	**1**	1	**2**	**2**	2	**2**	2	2	**2**

**Table 4:** Substitution, insertion and deletion costs for edges proposed by Harper et al. [33].

**Substitution Costs**			
**For Edges**			
	**-**	**=**	**≡**
**-**	0	3	3
**=**	3	0	3
**≡**	3	3	0
**Insertion/Deletion Costs**			
**For Edges**			
	**-**	**=**	**≡**
insert	0	**1**	1
delete	0	**1**	1

**Table 5:** Costs obtained in [35]. Each row corresponds to one of their experiments.

	Type of Cost	CAPST	DUD-E	GLL&GDD	MUV	NRLiSt_BDB	ULS-UDS
C1	Ins/Del [6]	0.000002	0.005	0.014	0.490	0.012	0.115
C2	Subs [5] by [6]	0.013	0.145	0.333	0.867	0.104	0.500
C3	Ins/Del [-]	0.004	0.001	0.003	0.327	0.003	0.011
C4	Subs [-] by [=]	0.017	0.186	0.206	1.005	0.024	0.607

**Table 6:** Accuracy (%) obtained in each dataset. In bold, the highest ones. The last column shows the mean accuracy.

	CAPST	DUD-E	GLL&GDD	MUV	NRLiSt_BDB	ULS-UDS	Mean
**Harper**	93.75	95.88	85.68	**92.76**	93.17	**96.10**	92.89
**1s**	92.93	91.25	93.03	56.01	94.75	92.94	86.82
**C1**	89.25	92.63	82.47	86.06	88.58	89.65	88.11
**C2**	89.75	91.13	82.51	87.35	88.21	91.69	88.44
**C3**	91.25	91.25	83.25	86.65	87.75	92.34	88.75
**C4**	89.50	90.88	82.43	86.00	89.92	92.59	88.55
**Our method**	**95.85**	**96.38**	**93.67**	88.63	**95.90**	94.00	**94.07**
**(Harper init.)**							
**Our method**	88.15	93.50	93.30	61.76	94.98	95.25	87.82
**(1s init.)**							

**Table 7:** Substitution, insertion and deletion costs of nodes obtained with our method. In bold, the ones that are different from Table 3 and Table 4.

**Substitution Costs for Nodes**															
	**[0]**	**[1]**	**[2]**	**[3]**	**[4]**	**[5]**	**[6]**	**[7]**	**[0, 1]**	**[0, 2]**	**[0, 3]**	**[1, 2]**	**[1, 3]**	**[2, 3]**	**[0, 1, 2]**
**[0]**	0	**1.99**	**2.02**	2.00	**1.99**	**2.04**	**2.05**	3.00	**1.06**	**0.99**	1.00	2.00	2.00	2.00	**0.97**
**[1]**	**1.99**	0	2.00	2.00	**1.98**	**1.99**	**1.96**	3.00	**1.02**	**1.99**	2.00	**1.02**	1.00	2.00	**1.04**
**[2]**	**2.02**	2.00	0	2.00	2.00	2.00	**1.99**	3.00	2.00	**0.99**	2.00	1.00	2.00	1.00	**0.98**
**[3]**	2.00	2.00	2.00	0	2.00	2.00	**2.05**	3.00	**1.99**	2.00	1.00	2.00	1.00	1.00	2.00
**[4]**	**1.99**	**1.98**	2.00	2.00	0	**1.99**	**2.01**	3.00	**2.01**	**2.01**	2.00	2.00	2.00	2.00	2.00
**[5]**	**2.04**	**1.99**	2.00	2.00	**1.99**	0	**1.99**	3.00	**1.96**	**1.96**	2.00	2.00	2.00	2.00	**2.02**
**[6]**	**2.05**	**1.96**	**1.99**	**2.05**	**2.01**	**1.99**	0	3.00	2.00	**2.01**	2.00	2.00	2.00	2.00	**1.98**
**[7]**	3.00	3.00	3.00	3.00	3.00	3.00	3.00	0	3.00	3.00	3.00	3.00	3.00	3.00	3.00
**[0, 1]**	**1.06**	**1.02**	2.00	**1.99**	**2.01**	**1.96**	2.00	3.00	0	**2.02**	2.00	2.00	2.00	2.00	**2.01**
**[0, 2]**	**0.99**	**1.99**	**0.99**	2.00	**2.01**	**1.96**	**2.01**	3.00	**2.02**	0	2.00	2.00	2.00	2.00	2.00
**[0, 3]**	1.00	2.00	2.00	1.00	2.00	2.00	2.00	3.00	2.00	2.00	0	2.00	2.00	2.00	2.00
**[1, 2]**	2.00	**1.02**	1.00	2.00	2.00	2.00	2.00	3.00	2.00	2.00	2.00	0	2.00	2.00	2.00
**[1, 3]**	2.00	1.00	2.00	1.00	2.00	2.00	2.00	3.00	2.00	2.00	2.00	2.00	0	2.00	2.00
**[2, 3]**	2.00	2.00	1.00	1.00	2.00	2.00	2.00	3.00	2.00	2.00	2.00	2.00	2.00	0	2.00
**[0, 1, 2]**	**0.97**	**1.04**	**0.98**	2.00	2.00	**2.02**	**1.98**	3.00	**2.01**	2.00	2.00	2.00	2.00	2.00	0
**Insertion/Deletion Costs for Nodes**															
	**[0]**	**[1]**	**[2]**	**[3]**	**[4]**	**[5]**	**[6]**	**[7]**	**[0, 1]**	**[0, 2]**	**[0, 3]**	**[1, 2]**	**[1, 3]**	**[2, 3]**	**[0, 1, 2]**
**insert**	**1.95**	**1.98**	2.00	2.00	**1.99**	**1.89**	**0.97**	1.00	**2.03**	**2.02**	2.00	**1.99**	2.00	2.00	**1.96**
**delete**	**1.95**	**1.98**	2.00	2.00	**1.99**	**1.89**	**0.97**	1.00	**2.03**	**2.02**	2.00	**1.99**	2.00	2.00	**1.96**

**Table 8:** Substitution, insertion and deletion costs of edges obtained with our method.

**Substitution Costs**			
**For Edges**			
	**-**	**=**	**≡**
**-**	0	3.00	3.00
**=**	3.00	0	3.00
**≡**	3.00	3.00	0
**Insertion/Deletion Costs**			
**For Edges**			
	**-**	**=**	**≡**
insert	0	**1.02**	1.00
delete	0	**1.02**	1.00
